# Impaired Mitochondrial Mobility in Charcot-Marie-Tooth Disease

**DOI:** 10.3389/fcell.2021.624823

**Published:** 2021-02-01

**Authors:** Cara R. Schiavon, Gerald S. Shadel, Uri Manor

**Affiliations:** ^1^Waitt Advanced Biophotonics Center, Salk Institute for Biological Studies, La Jolla, CA, United States; ^2^Molecular and Cell Biology Laboratory, Salk Institute for Biological Studies, La Jolla, CA, United States

**Keywords:** organelle transport, axonal transport deficiency, neurodegeneration, cytoskeleton, mitochondria, Charcot-Marie-Tooth (CMT) disease

## Abstract

Charcot-Marie-Tooth (CMT) disease is a progressive, peripheral neuropathy and the most commonly inherited neurological disorder. Clinical manifestations of CMT mutations are typically limited to peripheral neurons, the longest cells in the body. Currently, mutations in at least 80 different genes are associated with CMT and new mutations are regularly being discovered. A large portion of the proteins mutated in axonal CMT have documented roles in mitochondrial mobility, suggesting that organelle trafficking defects may be a common underlying disease mechanism. This review will focus on the potential role of altered mitochondrial mobility in the pathogenesis of axonal CMT, highlighting the conceptional challenges and potential experimental and therapeutic opportunities presented by this “impaired mobility” model of the disease.

## Introduction

Charcot-Marie-Tooth (CMT) disease is the most commonly inherited neurological disorder, affecting ∼1 in 5000 people (Skre, 1974; Barreto et al., 2016). It is a peripheral neuropathy defined by progressive deterioration of the peripheral nerves in the distal parts of the body, specifically the feet, hands, and lower extremities. This typically results in both motor and sensory loss in the affected areas. Unraveling the pathogenic mechanism(s) underlying CMT is somewhat complicated by the fact that CMT is both genetically and clinically heterogeneous. CMT has many subtypes including, demyelinating (affecting mainly Schwann cells), axonal, and intermediate (affecting both axons and Schwann cells). Herein, we are focusing on perturbations of mitochondrial mobility that might underlie the pathogenesis of axonal CMT.

Charcot-Marie-Tooth variants were originally classified based purely on clinical data. However, a recent explosion in genetic data can be mined to generate some compelling hypotheses. To date, over 100 mutations across more than 40 different proteins have been implicated in axonal and intermediate CMT. A large fraction of CMT-associated proteins has been shown or is predicted to affect the mobility of mitochondria or other organelles (Table 1). In this review, we will focus on those that affect mitochondrial mobility and hypothesize that defects in this process might begin to explain why CMT mutations mainly affect peripheral neurons. At the same time, we highlight important limitations to this “impaired mobility” model. Our belief is that the insights gained from studying the effects of CMT mutations in peripheral neurons will inform the role of mitochondrial mobility in other types of neurons and neurodegenerative disorders, including those associated with aging.

**TABLE 1:** Genes mutated in axonal and intermediate CMT.

**Gene/CMT subtype/OMIM Code**	**Function**	**References**
AARS1/CMT2N/613287	Catalyzes the attachment of alanine to tRNA.	McLaughlin et al., 2012; Zhao Z. et al., 2012; Bansagi et al., 2015; Dohrn et al., 2017; Weterman et al., 2018
ATP1A1/CMT2DD/618036	Catalyzes the hydrolysis of ATP coupled with the exchange of sodium and potassium ions across the plasma membrane.	Lassuthova et al., 2018
BAG3/CMT2*	Acts as a nucleotide-exchange factor promoting the release of ADP from the HSP70 and HSC70 proteins thereby triggering client/substrate protein release. Has anti-apoptotic activity. Plays a role in cytoskeletal proteostasis and dynamics.	Kim et al., 2018; Shy et al., 2018
BSCL2/CMT2/619112*	Plays a crucial role in the formation of lipid droplets. Mediates the formation and/or stabilization of endoplasmic reticulum-lipid droplet contacts. Binds anionic phospholipids including phosphatidic acid.	Chaudhry et al., 2013
DCTN2/CMT2*	Component of a large macromolecular complex required for the cytoplasmic dynein-driven movement of organelles along microtubules. Pays a role in prometaphase chromosome alignment and spindle organization during mitosis.	Braathen et al., 2016
DGAT2/CMT2	Required for synthesis and storage of intracellular triglycerides.	Hong et al., 2016
DHTKD1/CMT2Q/615025	Catalyzes the overall conversion of 2-oxoglutarate to succinyl-CoA and CO_2_.	Baets et al., 2014; Dohrn et al., 2017; Zhao et al., 2019
DNAJB2/CMT2T/604139	Functions as a co-chaperone, activating the ATPase activity of chaperones of the HSP70/heat shock protein 70 family. Contributes to the ubiquitin-dependent proteasomal degradation of misfolded proteins.	Gess et al., 2014; Lupo et al., 2016
DNM2/CMT2M/602378; CMTDIB/606482*	Plays an important role in vesicular trafficking processes, in particular endocytosis. Involved in producing microtubule bundles. Involved in cytokinesis.	Echaniz-Laguna et al., 2007; Bitoun et al., 2008; Haberlová et al., 2011; González-Jamett et al., 2014; Saghira et al., 2018
DYNC1H1/CMT2O/614228*	Acts as a motor for the intracellular retrograde motility of vesicles and organelles along microtubules. Plays a role in mitotic spindle assembly and metaphase plate congression.	Weedon et al., 2011
GARS1/CMT2D/601472	Catalyzes the attachment of glycine to tRNA	James et al., 2006; Xie et al., 2006; Hamaguchi et al., 2010; Motley et al., 2011; Morelli et al., 2017
GDAP1/CMT2K/607831; CMTRIA608340/; CMT4A/214400*	Regulates the mitochondrial network by promoting mitochondrial fission. Proposed roles in mitochondrial transport, redox processes, calcium homeostasis, and energy production.	Baxter et al., 2002; Cuesta et al., 2002; Ammar et al., 2003; Senderek et al., 2003; Di Maria et al., 2004; Zhang et al., 2004; Baránková et al., 2007; Kabzińska et al., 2007; Auer-Grumbach et al., 2008; Rougeot et al., 2008; Xin et al., 2008; Moroni et al., 2009; Sahin-Calapoglu et al., 2009a,b; Cassereau et al., 2011; Fusco et al., 2011; Zimoń et al., 2011, 2015; Vital et al., 2012; Auranen et al., 2013; Manganelli et al., 2015; Martin et al., 2015; Dohrn et al., 2017; Ho et al., 2017; Martí et al., 2017; Yoshimura et al., 2017; He et al., 2018; Masingue et al., 2018; Rzepnikowska and Kochański, 2018; Mai et al., 2019; Qin et al., 2019
HARS1/CMT2W/616625	Catalyzes the attachment of histidine to tRNA.	Baets et al., 2014
HINT1/CMT2/137200	Hydrolyzes purine nucleotide phosphoramidates with a single phosphate group.	Baets et al., 2014; Laššuthová et al., 2015; Zimoń et al., 2015; Dohrn et al., 2017
HSPB1/CMT2F/606595*	Functions as a molecular chaperone maintaining denatured proteins in a folding-competent state. Plays a role in stress resistance and actin organization. Regulates numerous biological processes including phosphorylation and axonal transport of neurofilament proteins.	Liu et al., 2005; Tang et al., 2005; Chung et al., 2008; Houlden et al., 2008; Solla et al., 2010; Amornvit et al., 2017; Dohrn et al., 2017; Ho et al., 2017; Weeks et al., 2018
HSPB8/CMT2L/608673*	Displays temperature-dependent chaperone activity. Forms complex with BAG3.	Nicholson et al., 2009
IGHMBP2/CMT2S/616155	5′ to 3′ helicase that unwinds RNA and DNA duplices. Acts as a transcription regulator.	Cottenie et al., 2014; Wagner et al., 2015; Dohrn et al., 2017; Yuan et al., 2017
JPH1/CMT2K/607831	Contributes to the formation of junctional membrane complexes which link the plasma membrane with the endoplasmic or sarcoplasmic reticulum in excitable cells. Provides a structural foundation for functional cross-talk between the cell surface and intracellular calcium release channels.	Pla-Martín et al., 2015; Kanwal and Perveen, 2019
KIF1B/CMT2A1/118210*	Motor for anterograde transport of mitochondria.	Nakagawa and Takashima, 2003; Bissar-Tadmouri et al., 2004
KIF5A/CMT2*	Microtubule-dependent motor required for slow axonal transport of neurofilament proteins. Contributes to the vesicular transport of VAPA, VAPB, SURF4, RAB11A, RAB11B and RTN3 proteins in neurons.	Dohrn et al., 2017
LMNA/CMT2B1/605588	Plays an important role in nuclear assembly, chromatin organization, nuclear membrane and telomere dynamics.	De Sandre-Giovannoli et al., 2002; Chaouch et al., 2003; Hamadouche et al., 2008; Zhang et al., 2010; Liang et al., 2016; Dohrn et al., 2017
LRSAM1/CMT2G/614436	E3 ubiquitin-protein ligase. Bacterial recognition protein that defends the cytoplasm from invasive pathogens. Potential role in mitophagy?	Guernsey et al., 2010; Nicolaou et al., 2013; Dohrn et al., 2017
MARS1/CMT2U/616280	Catalyzes the attachment of methionine to tRNA.	Baets et al., 2014; Sun et al., 2017
MED25/CMT2B2/605589	A coactivator involved in the regulated transcription of nearly all RNA polymerase II-dependent genes.	Leal et al., 2009, 2018; Tazir et al., 2013
MFN2/CMT2A2/609260*	Mitochondrial outer membrane GTPase that mediates mitochondrial clustering and fusion. Involved in the clearance of damaged mitochondria via mitophagy. Potential roles in mitochondria-ER contacts and mitochondrial transport.	Kijima et al., 2005; Lawson et al., 2005; Engelfried et al., 2006; Verhoeven et al., 2006; Zuchner et al., 2006; Loiseau et al., 2007; Muglia et al., 2007; Neusch et al., 2007; Del Bo et al., 2008; Calvo et al., 2009; Cartoni and Martinou, 2009; Braathen et al., 2010; Ouvrier and Grew, 2010; Feely et al., 2011; McCorquodale et al., 2011; Park et al., 2012; Vital et al., 2012; Brožková et al., 2013; Chapman et al., 2013; Kotruchow et al., 2013; Lv et al., 2013, 2015; Vielhaber et al., 2013; Bergamin et al., 2014; Choi B. O. et al., 2015; Wang et al., 2015; Bannerman et al., 2016; Di Meglio et al., 2016; Neupauerová et al., 2016; Rudnik-Schöneborn et al., 2016; Tan et al., 2016; Werheid et al., 2016; Xie et al., 2016; Ando et al., 2017; Beręsewicz et al., 2017; Dohrn et al., 2017; El Fissi et al., 2018; Finsterer et al., 2018; Iapadre et al., 2018; Milley et al., 2018; Larrea et al., 2019; Xu et al., 2019
MME/CMT2T/617017	Biologically important in the destruction of opioid peptides. Able to cleave angiotensin. Involved in the degradation of atrial natriuretic factor.	Auer-Grumbach et al., 2016; Fujisawa et al., 2017
MORC2/CMT2Z/616688	Essential for epigenetic silencing by the HUSH complex.	Albulym et al., 2016
MPV17/CMT2EE/618400*	Non-selective channel that modulates membrane potential under normal conditions and oxidative stress, and is involved in mitochondrial homeostasis. Involved in mitochondrial deoxynucleoside triphosphates pool homeostasis and mitochondrial DNA maintenance.	Choi Y. R. et al., 2015
MPZ/CMT2J/607736; CMTDID/607791; CMT1B/118200	Mediates adhesion between adjacent myelin wraps and ultimately drives myelin compaction.	Hayasaka et al., 1993; Pham-Dinh et al., 1993; Nelis et al., 1994; Latour et al., 1995; Blanquet-Grossard et al., 1996; Roa et al., 1996; Silander et al., 1996; Bissar-Tadmouri et al., 1999; De Jonghe et al., 1999; Lagueny et al., 1999; Quattrini et al., 1999; Senderek et al., 2000; Kochański et al., 2004; Kurihara et al., 2004; Bienfait et al., 2006; Sabet et al., 2006; Laurà et al., 2007; Lee et al., 2008; Mazzeo et al., 2008; Gallardo et al., 2009; Avila et al., 2010; Brozková et al., 2010; Kleffner et al., 2010; Choi et al., 2011; Høyer et al., 2011, 2014; Chavada et al., 2012; Maeda et al., 2012; Marttila et al., 2012; Rosberg et al., 2013; Speevak and Farrell, 2013; Bergamin et al., 2014; Leal et al., 2014; Sanmaneechai et al., 2015; Tokuda et al., 2015; Wang et al., 2015; Rudnik-Schöneborn et al., 2016; Werheid et al., 2016; Dohrn et al., 2017; He et al., 2018; Milley et al., 2018; Xu et al., 2019
MT-ATP6/CMT2*	Mitochondrial membrane ATP synthase. Key component of the proton channel.	Pitceathly et al., 2012
MYH14/CMT2/614369*	Conventional non-muscle myosin. Actin-dependent motor protein. Mediates mitochondrial fission.	Almutawa et al., 2019
NAGLU/CMT2V/616491	Involved in the degradation of heparan sulfate.	Tétreault et al., 2015
NEFH/CMT2CC/616924*	Component of neurofilaments, the most abundant cytoskeletal component of myelinated axons.	Bian et al., 2018
NEFL/CMT2E/607684*	Component of neurofilaments, the most abundant cytoskeletal component of myelinated axons. Regulates mitochondrial morphology.	Lupski, 2000; Luo et al., 2003; Fabrizi et al., 2007; Miltenberger-Miltenyi et al., 2007; Shin et al., 2008; Bhagavati et al., 2009; Berciano et al., 2016; Werheid et al., 2016; Dohrn et al., 2017; Horga et al., 2017; Fu and Yuan, 2018; Xu et al., 2019
PNKP/CMT2B2/605589	Plays a key role in the repair of DNA damage, functioning as part of both the non-homologous end-joining and base excision repair pathways.	Leal et al., 2018
RAB7A/CMT2B/600882*	Key regulator in endo-lysosomal trafficking. Plays roles in growth-factor-mediated cell signaling, nutrient-transporter mediated nutrient uptake, neurotrophin transport in the axons of neurons and lipid metabolism. Regulates mitochondrial fission, mitophagy, and mitochondria-lysosome tethering.	Meggouh et al., 2006; Zhang et al., 2010; Manganelli et al., 2015
SPG11/CMT2X/616668*	Plays a role in neurite plasticity by maintaining cytoskeleton stability and regulating synaptic vesicle transport.	Montecchiani et al., 2016
TRIM2/CMT2R/615490*	E3 ubiquitin-protein ligase that mediates the ubiquitination of NEFL and of phosphorylated BCL2L11.	Baets et al., 2014; Pehlivan et al., 2015
TRPV4/CMT2C/606071*	Non-selective calcium permeant cation channel involved in osmotic sensitivity and mechanosensitivity. Some data supporting a role in regulating mitochondrial motility.	Klein et al., 2003; Deng et al., 2010; Manganelli et al., 2015; Dohrn et al., 2017
VCP/CMT2Y/616687*	Necessary for the fragmentation of Golgi stacks during mitosis and for their reassembly after mitosis. Involved in the formation of the transitional endoplasmic reticulum. Plays a role in the regulation of stress granule clearance. Involved in DNA damage response. Essential for the maturation of ubiquitin-containing autophagosomes and the clearance of ubiquitinated protein by autophagy and mitophagy.	Gonzalez et al., 2014
C1ORF194/CMTDI	May affect intracellular Ca^2+^ homeostasis.	Sun et al., 2019
GNB4/CMTDIF/615185	Modulator/transducer in various transmembrane signaling systems.	Soong et al., 2013; Baets et al., 2014; Miura et al., 2017
INF2/CMTDIE/614455*	Mediates actin polymerization at ER-mitochondria contact sites. Regulates mitochondrial morphology and motility.	Boyer et al., 2011a,b; Mademan et al., 2013; Rodriguez et al., 2013; Vallat et al., 2013; Caridi et al., 2014; Park et al., 2014; Jin et al., 2015; Werheid et al., 2016; Dohrn et al., 2017; Echaniz-Laguna and Latour, 2019; Fu et al., 2019
SLC12A6/CMTDI/218000	Mediates electroneutral potassium-chloride cotransport.	Lupo et al., 2016
YARS1/CMTDIC/608323	Catalyzes the attachment of tyrosine to its corresponding tRNA	Jordanova et al., 2006; Xie et al., 2007
COX6A1/CMTRI/616039*	A subunit of the cytochrome c oxidase complex	Tamiya et al., 2014
KARS1/CMTRIB/613641	Catalyzes the aminoacylation of tRNA-Lys in the cytoplasm and mitochondria	McLaughlin et al., 2010
PLEKHG5/CMTRIC/615376	Activates the nuclear factor kappa B (NFKB1) signaling pathway. Also implicated in distal spinal muscular atrophy.	Kim et al., 2013

*Genes that play a role in mitochondrial function and/or motility are marked with an asterisk.*

## CMT Is a Progressive Disorder That Affects Predominantly the Longest Neurons

Charcot-Marie-Tooth usually affects only the feet, hands, and lower extremities. The axons leading to these distal sites can be as long as a meter in some individuals. There have been some reports of central nervous system involvement but these instances are rare (Pareyson and Marchesi, 2009; Lee et al., 2017). Some CMT mutations also cause optic atrophy, and the optical nerve notably consists of relatively long axons (∼50 mm). Patients are usually born unaffected, but typically by age 10 display major losses of function. However, the range of age can be from the toddler years to the 5th decade of life (Skre, 1974; Verhoeven et al., 2003; Zuchner et al., 2004, 2006; Chung et al., 2006; Engelfried et al., 2006; Cho et al., 2007; Calvo et al., 2009; Braathen et al., 2010; Boyer et al., 2011b). The severity of the disease is directly correlated with the age of onset (Chung et al., 2006; Verhoeven et al., 2006), and the longer axons (i.e., the feet) invariably degenerate before the shorter axons (i.e., the hands). In cases where CMT patients also display optic atrophy, this occurs after loss of function in the hands (Verhoeven et al., 2006; Zuchner et al., 2006). Together, these observations indicate a disease that directly correlates the length of the axon with the speed of onset, and the speed of onset with the magnitude of the pathology.

## CMT Mutations Largely Affect Mitochondrial Mobility

As mentioned above, a unique peripheral nerve characteristic is their extreme length, which suggests these cells are uniquely sensitive to impaired long-distance transport. Put simply, a decrease in mobility would have a greater impact on longer distance commutes than shorter ones. In support of this theory, 23 out of the 48 genes mutated in axonal or intermediate CMT encode proteins that play roles in mitochondrial function, often impacting mitochondrial mobility (Table 1).

The majority of axonal CMT studies have centered on Mitofusin 2 (MFN2) mutations, which consistently result in reduced axonal mitochondrial mobility. This phenotype has been reproduced in mouse models and in patient cell lines and tissues. Neurons expressing MFN2 CMT mutants and neurons from MFN2 CMT mouse models also show reduced axonal mitochondrial mobility (Baloh et al., 2007; Vallat et al., 2008; Rocha et al., 2018). MFN2 is also implicated in mitochondrial fusion dynamics, and MFN2 CMT mutations cause clustering of improperly fused mitochondria (Baloh et al., 2007; Detmer and Chan, 2007; Vallat et al., 2008; Rocha et al., 2018). Thus, it is possible that this mitochondrial clustering contributes to reduced mitochondrial mobility. Although several MFN2 CMT mutants cause mitochondrial fragmentation suggesting a disruption of its fusogenic activity, there are other MFN2 CMT mutants that do not alter mitochondrial morphology or, seemingly paradoxically, even cause mitochondrial elongation (Detmer and Chan, 2007; Codron et al., 2016; Rocha et al., 2018). MFN2 is also implicated in mitophagy, lipid transfer, lipid droplet-mitochondria contacts, and endoplasmic reticulum (ER)-mitochondria contacts, although whether MFN2 increases or decreases ER-mitochondria contacts is still under debate (de Brito and Scorrano, 2008; Chen and Dorn, 2013; Sugiura et al., 2013; Gong et al., 2015; Leal et al., 2016; Naon et al., 2016, 2017; Boutant et al., 2017; Filadi et al., 2017; Basso et al., 2018; McLelland et al., 2018; Hernández-Alvarez et al., 2019). While MFN2 CMT mutants reduce ER-mitochondria contacts (Bernard-Marissal et al., 2019; Larrea et al., 2019), it is unclear whether these changes affect mitochondrial mobility.

How alterations in mitochondrial motility impact mitochondrial function, particularly in the context of CMT, remains poorly understood. Despite clear defects in mitochondrial mobility, some studies have concluded CMT mutations do not alter readouts of mitochondrial OXPHOS function such as mitochondrial membrane potential, oxygen consumption, and ATP production, or impair cellular calcium levels which mitochondria are involved in controlling (Baloh et al., 2007; Larrea et al., 2019). However, other studies have demonstrated that CMT mutations cause defects in these readouts (Loiseau et al., 2007; Guillet et al., 2010; Barneo-Munoz et al., 2015; Saporta et al., 2015; Rocha et al., 2018; Almutawa et al., 2019; Bernard-Marissal et al., 2019). And, another study demonstrated that bioenergetic efficiency and viability in a fly model can be rescued with only minor alterations in mitochondrial distribution (Trevisan et al., 2018). These discrepancies may be at least partially explained by differences in the model systems and experimental conditions used. There are now a wide variety of tools to study CMT including mouse and fly (*Drosophila melanogaster*) genetic models and iPSC-derived motor neurons (Saporta et al., 2015; Yamaguchi and Takashima, 2018; Juneja et al., 2019).

There is also clear evidence for a role of organelle-organelle contacts affecting mitochondrial mobility in CMT caused by mutations in the endo-lysosomal protein RAB7A. Wong et al. demonstrated reduced mitochondrial mobility due to prolonged inter-mitochondrial contacts in HeLa cells expressing CMT-mutant MFN2, RAB7A, or TRPV4 (Transient Receptor Potential Cation Channel Subfamily V Member 4) (Wong et al., 2019). RAB7A CMT mutations also increase tethering between mitochondria and endolysosomes, leading to changes in mitochondrial morphology and reduced mitochondrial mobility (Wong et al., 2018, 2019; Cioni et al., 2019). There is also evidence pointing towards an interaction between RAB7A and MFN2 (Zhao T. et al., 2012). Together, these findings suggest that interpretations of RAB7A mutations causing CMT based solely on defects in its endo-lysosomal function may be too simplistic. In the same vein, a recent study found that CMT-causing GDAP1 (Ganglioside Induced Differentiation Associated Protein 1) mutations result in defective mitochondria-lysosome contacts (Cantarero et al., 2020). That mitochondria-organelle contacts can affect mitochondrial mobility and dynamics highlights the limitations of evaluating protein and organelle dysfunction in isolation.

Recently, a screen for RAB7A binding partners found that another CMT protein, INF2 (Inverted Formin 2), is one of several actin-binding candidate interaction partners for RAB7A (Pan et al., 2020). This is particularly relevant to our discussion on CMT, inter-organelle contacts, and mitochondrial mobility for multiple reasons. First, a splice isoform of INF2 is tail-anchored to the ER. Second, dominant active mutations in ER-anchored INF2 that mimic INF2 CMT mutations have been shown to increase actin-dependent mitochondrial fragmentation and decrease mitochondrial mobility (Korobova et al., 2013; Chakrabarti et al., 2018). Together, these data point towards an important role in ER-mitochondria inter-organelle contacts in somehow regulating mitochondrial mobility via the actin cytoskeleton. That INF2 also potentially interacts with RAB7A suggests that mitochondria, endo-lysosomes, and ER all contact one another via CMT-associated proteins.

**FIGURE 1:**
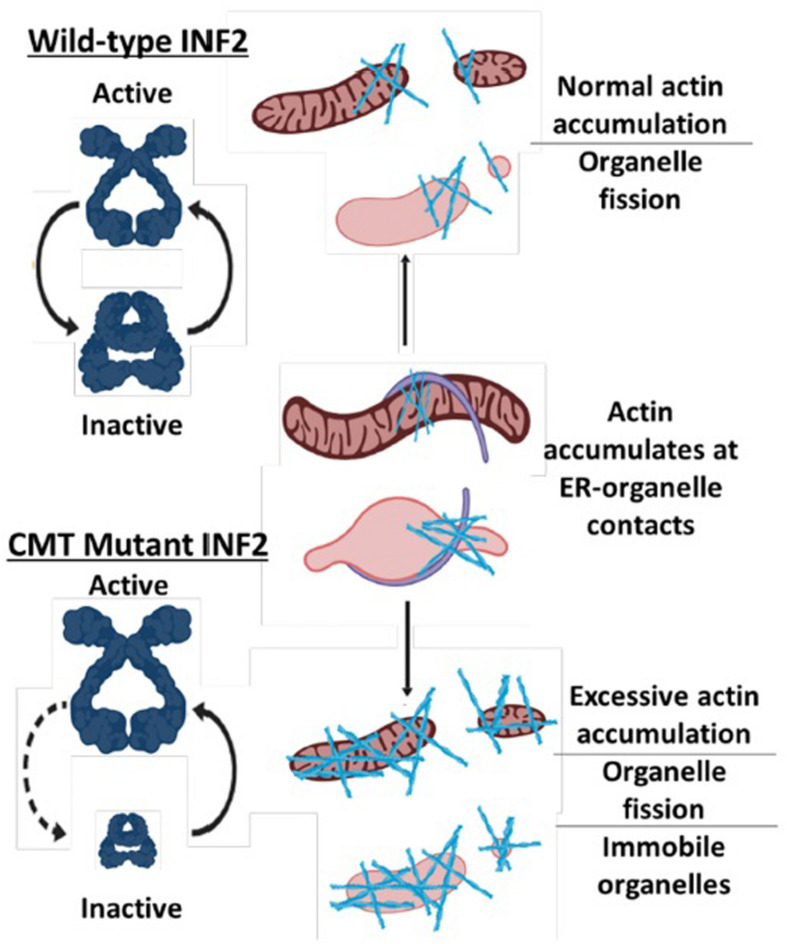
Model for how CMT mutations in INF2 reduce mitochondrial mobility. Normal INF2 is autoinhibited as indicated by open conformation compared to the closed conformation and thus has regulated actin-assembly activity. CMT mutations in INF2 reduce autoinhibition, resulting in excessive actin assembly on mitochondria and potentially other organelles, which in turn reduces their mobility.

All INF2 CMT mutations are predicted or have been shown to increase actin assembly (Bayraktar et al., 2020). While some actin-binding motor proteins likely facilitate microtubule-independent mitochondrial transport, numerous studies have shown that long-range microtubule-based mobility of mitochondria is antagonized by actin and actin-binding motor proteins (Chada and Hollenbeck, 2004; Quintero et al., 2009; Pathak et al., 2010; Venkatesh et al., 2019; Cardanho-Ramos et al., 2020). Thus, while the effects of INF2 CMT mutations have yet to be studied in neurons, it is reasonable to expect that INF2 CMT mutations will cause an actin-dependent decrease in mitochondrial mobility in axons. Furthermore, since the ER regularly contacts many other organelles, and even appears to drive actin-assembly at ER-organelle contact sites (Korobova et al., 2013, 2014; Manor et al., 2015; Chakrabarti et al., 2018; Yang and Svitkina, 2019; Schiavon et al., 2020), it is quite possible INF2 CMT mutations cause aberrant actin assembly on other organelles, reducing their mobility as well (Figure 1).

Together with the newly uncovered role for RAB7A in (indirectly) modulating actin assembly (Pan et al., 2020), these observations point towards a role for multiple CMT mutations causing aberrant organelle-organelle and organelle-actin contacts, all of which cause decreased mitochondrial mobility. Whether some (or all) CMT mutations also cause decreased mobility of other organelles remains an important open question.

The focus of the role of mitochondria in CMT has been primarily on MFN2 and GDAP1, and to a lesser extent on associated motor proteins (KIF1B – Kinesin Family Member 1B, KIF5A – Kinesin Family Member 5A, DYNC1H1 – Dynein Cytoplasmic 1 Heavy Chain 1, DCTN2 – Dynactin Subunit 2) and some cytoskeletal proteins (NEFL – Neurofilament Light). Here, we have highlighted INF2 and RAB7A as CMT-associated proteins likely involved in mitochondrial mobility and dynamics. However, we propose that the proteins mutated in CMT that play roles in mitochondrial function, dynamics and mobility likely extend well beyond just these two (see Table 1 for a full list).

## Why Do Mobility Defects Usually Only Affect Peripheral Neurons in CMT Patients?

Hopefully, we have provided a convincing argument that many CMT mutations likely reduce mitochondrial mobility. Given the extreme lengths of peripheral axons, it is tempting to conclude that a reduction in mobility due to CMT mutations simply affects longer axons more severely (the “impaired mobility model” of CMT). One can easily reconcile two key features of CMT using the impaired mobility model: The progressive nature of the disease: This suggests that dysfunction must accumulate over time. One can imagine this more severely affects longer axons, due to reduced turnover of damaged mitochondria resulting from reduced mitochondrial mobility. Interestingly, one could imagine that reduced mobility of other organelles associated with turnover (e.g., lysosomes) could also cause increased accumulation of damaged mitochondria in longer axons. The longest peripheral axons (i.e., the feet) progressively degenerate prior to the next-longest axons (i.e., the hands): This further supports the impaired mobility model, wherein damage accumulates first in the longest axons due to the more demanding, “longer commute” resulting in faster accumulation of damaged mitochondria.

**FIGURE 2:**
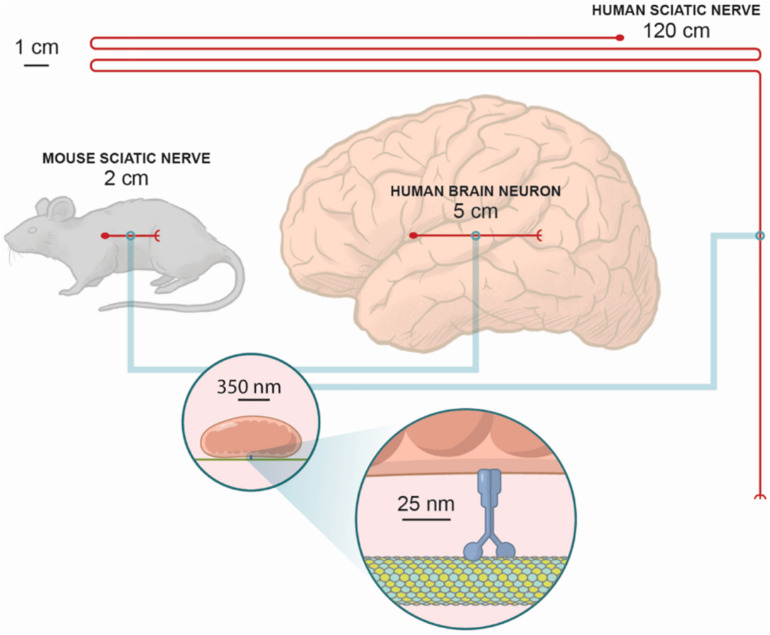
The relative scales of axons in humans versus mice. While microtubules, motor proteins, and mitochondria have nearly identical sizes in mice and men, the length of the axons can be very different. At the same time, axons in the mouse sciatic nerve are not as long as some of the longer axons within the human brain. For example, it is known in the macaque there are direct connections from the frontal eye fields in the anterior bank of the arcuate sulcus to the primary visual cortex, which in the macaque is ∼5 cm, and probably as long as 12.5 cm in humans. Notably, for most CMT patients the pathology is often constrained to only peripheral neurons. Since the same mutations can cause CMT in mice and men, the distances mitochondria must travel can only provide a partial explanation for the physiopathology of these mutations.

Unfortunately, while this model is compelling, it appears to be overly simplistic. The weakness in relying on mobility alone as an explanation can best be highlighted by comparing the lengths of different axons both within and between species. For example, the longest axon in mice is approximately 2 cm, whereas in humans the longest axon is ∼60 times longer. Just as striking, some unaffected axons in the human CNS may be longer than the mouse’s longest axon. The very same mutation in humans and mice can cause CMT, yet no defects are found in the brains of human CMT patients. Meanwhile, the motor proteins, cytoskeletal tracks, and mitochondria of mice and humans are all roughly the same size, and all possess roughly the same biophysical properties (e.g., velocity, force generation, etc.) when transporting their organelles across long distances (Figure 2).

Any analysis of mobility must consider not just distance but also time. Most laboratory mice only live for ∼2 years, while human CMT patients may not even experience symptoms until adolescence or adulthood. Thus, there is clearly a “missing variable” that underlies differences in lifespan and disease susceptibility between species (e.g., differences in metabolism or oxidative stress). Thus, CMT may serve as a “model disease” to better understand age-related neurodegeneration. It is well established mitochondria play myriad roles at the pre-synapse, including ATP production, intra- and intercellular signaling (e.g., calcium signaling and signaling via reactive oxygen species), and the biosynthesis of signaling molecules (e.g., lipids, hormones, and neurotransmitter intermediates) (Devine and Kittler, 2018). Perhaps perturbed transport of mitochondria to the pre-synapse of peripheral neurons in CMT provides an opportunity to better understand other neurodegenerative disorders associated with defective presynaptic mitochondria, including Alzheimer’s, ALS, Parkinson’s, Friedreich’s Ataxia, and Hereditary Spastic Paraplegia (Devine and Kittler, 2018).

But even considering the impaired mobility model for CMT within a single organism has some issues. It is difficult to imagine how reductions in mobility as high as 100% (Baloh et al., 2007; Rocha et al., 2018) could have a severe effect only on the longest axons, but not other axons that, while shorter than the peripheral neurons, are still very long compared to the ∼25 nm step size of a motor protein.

When considering these conundrums, it is helpful to consider alternative mechanisms for replenishing mitochondria in neurons, many of which are reviewed elsewhere (Misgeld and Schwarz, 2017; Yu and Pekkurnaz, 2018). Briefly, mitochondrial rejuvenation is speculated to be at least partially mediated via local translation in the axon. Interestingly, multiple CMT mutations affect local translation machinery (Table 1). More recent work showed that mitochondria serve as a stable compartment for mediating biogenesis by serving as an energy source for synaptic translation (Rangaraju et al., 2019). This raises a chicken vs. egg question: Does reduced mitochondrial mobility impair local translation needed for synaptic and therefore neuronal health and maintenance? Or does impaired local translation lead to dysfunctional mitochondria that cannot be replaced without sufficient mobility? That CMT is caused by mutations disrupting both mobility and local translation indicates these two processes have a unique relationship in long axons.

## Conclusion and Open Questions

One open question is how the overall distribution of mitochondria is altered in CMT neurons, and how this relates to axonal maintenance. A recent study showed mitochondria tend to distribute along the length of axons with regular spacing, and that inter-mitochondrial feedback mediates their positioning and movement (Matsumoto et al., 2020). Is this feedback-based positioning altered in CMT? Do mutations affecting mobility result in CMT via a “domino effect” caused by defects in relatively local repositioning between axonal mitochondria, which then cascades with increasing defects as a function of increasing axonal length? How much longer does it take mitochondria in CMT patients to traverse the entire length of an axon? Defective mitophagy has been implicated in other neurodegenerative disorders and some studies have linked CMT to alterations in autophagy (Colecchia et al., 2018; Gautam et al., 2019). Is there a reduction in the turnover rate of mitochondria in CMT patients? Mitochondria are increasingly being implicated as important players in adaptive and innate immune responses and inflammatory pathology, including neurodegeneration (West, 2017; Newman and Shadel, 2018). Could “mitoflammation” contribute to the pathophysiology of CMT? How do any and all of these factors affect mitochondria at the pre-synapse of CMT peripheral neurons, likely the most important subpopulation of mitochondria in these cells? These are surprisingly open questions we expect to be addressed in the coming years using animal and cell models of CMT.

## Data Availability Statement

The original contributions presented in the study are included in the article/supplementary material, further inquiries can be directed to the corresponding author.

## Author Contributions

All authors listed have made a substantial, direct and intellectual contribution to the work, and approved it for publication.

## Conflict of Interest

The authors declare that the research was conducted in the absence of any commercial or financial relationships that could be construed as a potential conflict of interest.
